# Domestication of captive-bred masu salmon *Oncorhynchus masou masou* (Salmonidae) leads to a significant decrease in numbers of lateral line organs

**DOI:** 10.1038/s41598-022-21195-3

**Published:** 2022-10-06

**Authors:** Masanori Nakae, Koh Hasegawa, Kouta Miyamoto

**Affiliations:** 1 Department of Zoology, National Museum of Nature and Science, 4-1-1 Amakubo, Tsukuba, Ibaraki 305-0005 Japan; 2 Salmon Research Department, Fisheries Resources Institute, Japan Fisheries Research and Education Agency, Nakanoshima, Toyohira, Sapporo 062-0922 Japan; 3 Nikko Field Station, Fisheries Technology Institute, Japan Fisheries Research and Education Agency, Nikko, Tochigi 321-1661 Japan

**Keywords:** Zoology, Ichthyology, Evolution

## Abstract

Because captive-bred animals gradually adapt to artificial rearing environments due to evolving life history traits, such individuals sometimes show lessened performance in natural environments. The lateral line system, one of the principal sensory organs of fishes, varies according to habitat environments, sometimes differing even within the same species. A reduction in lateral line elements may also occur in successive generations of captive-bred fish. Such a reduction, involving neuromasts over the entire body, was examined for the first time in captive-bred masu salmon *Oncorhynchus masou masou*. The total number of neuromasts in captive-bred fish was ca. 10% lower than in wild-caught and F1 fishes, suggesting that the system in captive-bred fish had reduced in number due to domestication. Furthermore, differences in total neuromast numbers between captive-bred and wild fish were greater than between anadromous and fluvial populations of the species. The lower number of neuromasts could be one of the reasons behind the lower survival of captive-bred fish in natural environments.

## Introduction

Captive breeding of animals in rearing facilities is a technique for producing individuals. They are sometimes stocked into natural environments for sustaining biological resources and conservation of endangered species^1–3^. However, such individuals suffer from domestication, gradually adapting to the captive breeding and artificial rearing environments through the evolution of life history traits^4^; those which are not important for adaptation to captivity may be lost through captive breeding^5,6^. Accordingly, captive-bred individuals sometimes perform poorly in natural environments, resulting in the failure of stocking programs^5^.

Sensory organs vary dramatically depending on environments, sometimes within the same species, an extreme example being the loss of sight in cave populations of the Mexican cavefish (*Astyanax mexicanus*)^7^. A reduction of sensory organs may also occur in artificial environments which lack predators and have adequate food supplied, since captive-bred individuals no longer need to be sensitive to the presence of predators and food items compared to individuals in natural environments^8^. However, in order to evaluate the degree of reduction of sensory organs, the latter must be considered in natural (wild) populations, since sensory organs sometimes show interpopulational variations^7^.

The lateral line system, one of the principal sensory organs of fishes, consists of canal (CN) and superficial (SN) neuromasts, CNs being located in subcutaneous canals, and SNs on the skin and scales. However, the functions of CNs and SNs more or less overlap, both mainly detecting relatively rapid hydrodynamic fluctuations, the former as a flow acceleration sensor, with low-pass filtering and more low-frequency signals (e.g., stream flow and current wakes), and the latter as a velocity sensor, with high-pass filtering^9^. Furthermore, the coverage of CNs and SNs on the head of many fish taxa, including salmonids, is broader than on the body, although some fishes have many SNs on the body (e.g., gobies^10^ and nurseryfishes^11^). The lateral line system also varies dramatically depending on habitat environments, sometimes differing within the same species (e.g., threespine sticklebacks^12^ and Mexican cavefish^13^), and may also be due to domestication, although not yet well clarified. In one of the few previous studies, Brown et al. (2013)^14^ demonstrated that captive-bred steelhead *Onchorhynchus mykiss* (Salmonidae), possessed significantly fewer SNs than wild individuals. However, because that study used wild and captive-bred fish that had originated from different populations, any interpopulational differences in the lateral line system due to the different origins were not ruled out.

In this study, we tested the hypothesis that neuromasts of captive-bred masu salmon (*O. masou masou*, a salmonid fish endemic to far eastern Asia), decreased in number from wild fish through domestication, by standardizing the origins of populations of experimental captive-bred and wild fishes. This is the first study which has considered different generations of captive-bred fish, as well as examining different body locations of both types of neuromast (i.e., CNs and SNs), so as to further understand the effects of domestication on the lateral line system.

## Results

**Figure 1:**
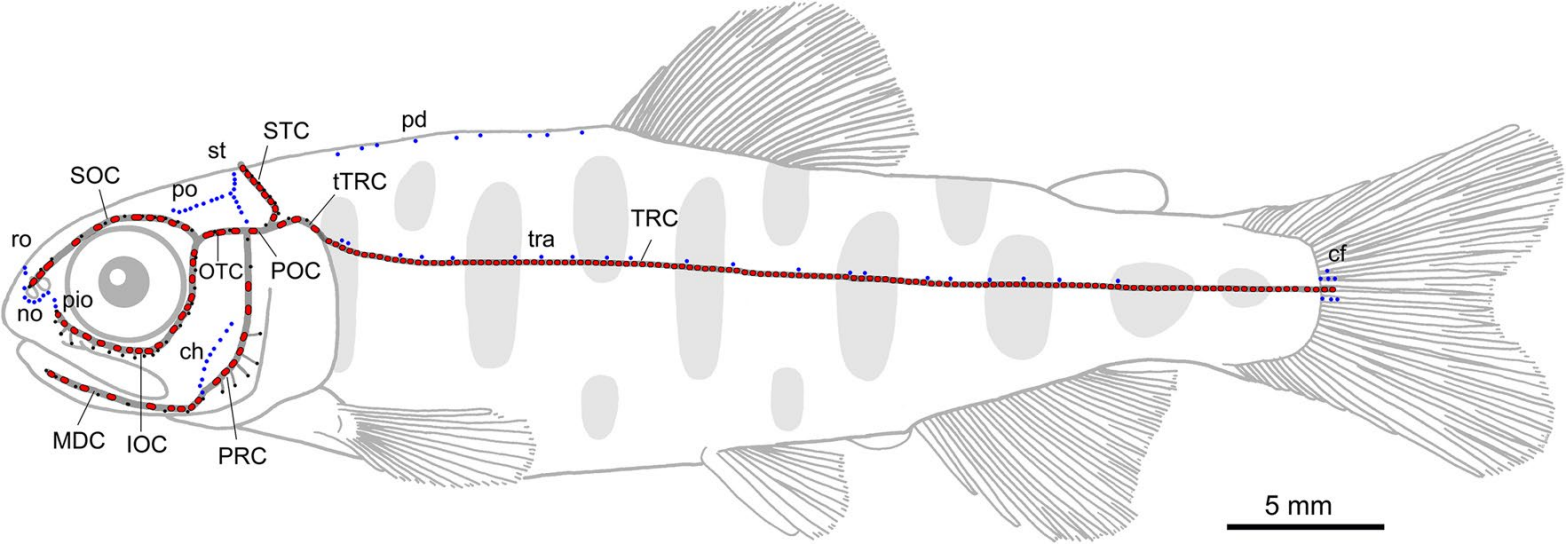
Lateral line system in *Oncorhynchus masou masou*. Dark grey, pores; grey, canals; large red dots, canal neuromasts; small blue dots, superficial neuromasts. Abbreviations of lateral line canals: *IOC* infraorbital canal, *MDC* mandibular canal, *OTC* otic canal, *POC* postotic canal, *PRC* preopercular canal, *SOC* supraorbital canal, *STC* supratemporal canal, *TRC* trunk canal, *tTRC* temporal portion of trunk canal. Abbreviations of superficial neuromasts groups (i.e., accessory lines): *cf* caudal fin, *ch* cheek, *no* nostril, *pd* predorsal, *pio* preinfraorbital, *po* postocular, *ro* rostral, *st* supratemporal, *tra* trunk accessory. Neuromasts of TRC, cf, pd and tra are categorized as those on the body. (after Nakae & Hasegawa 2022^22^: fig. 2; the figure was generated with Adobe Photoshop CC 2020).

**Figure 2:**
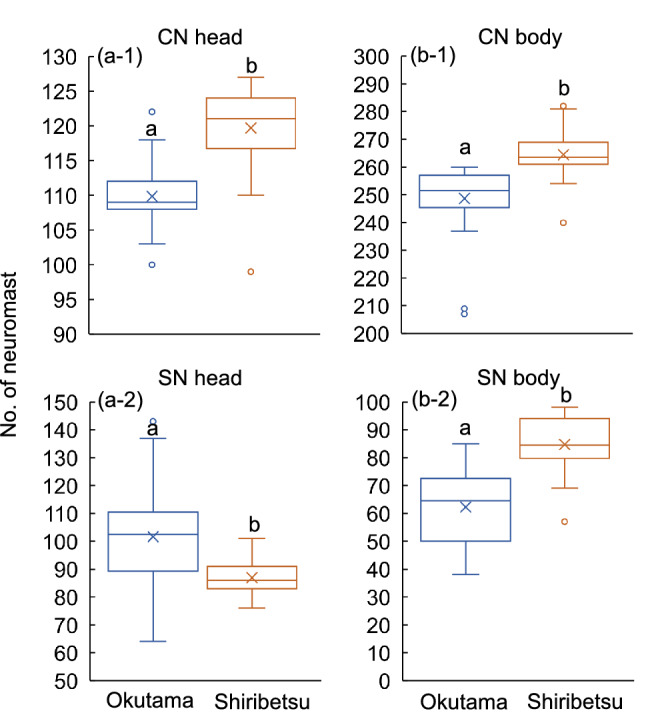
Comparisons of neuromast numbers (CNs and SNs) on the head and body of wild *Oncorhynchus masou masou* from Okutama and Shiribetsu regions. Different letters (a, b) indicate significant difference detected by univariate ANOVA (p < 0.05). Boxplots show 25, 50 and 75 percentiles, maximum and minimum values without considering outliers, and mean values (X).

**Figure 3:**
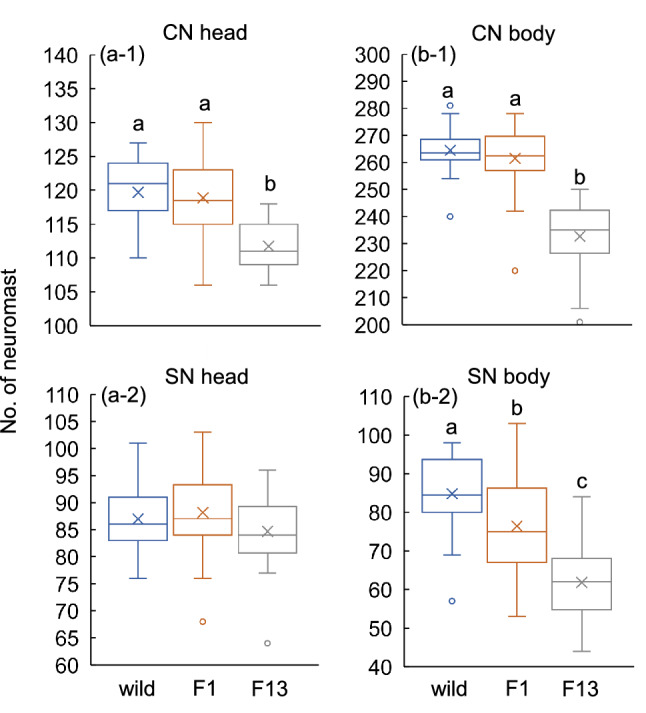
Comparisons of neuromast numbers (CNs and SNs) on the head and body of wild, F1 and F13 *Oncorhynchus masou masou* from the Shiribetsu anadromous population. Different letters (a, b, c) indicate significant differences detected by Scheffe’s test following univariate ANOVA (p < 0.05). Boxplots show 25, 50 and 75 percentiles, maximum and minimum values without considering outliers, and mean values (X).

**Figure 4:**
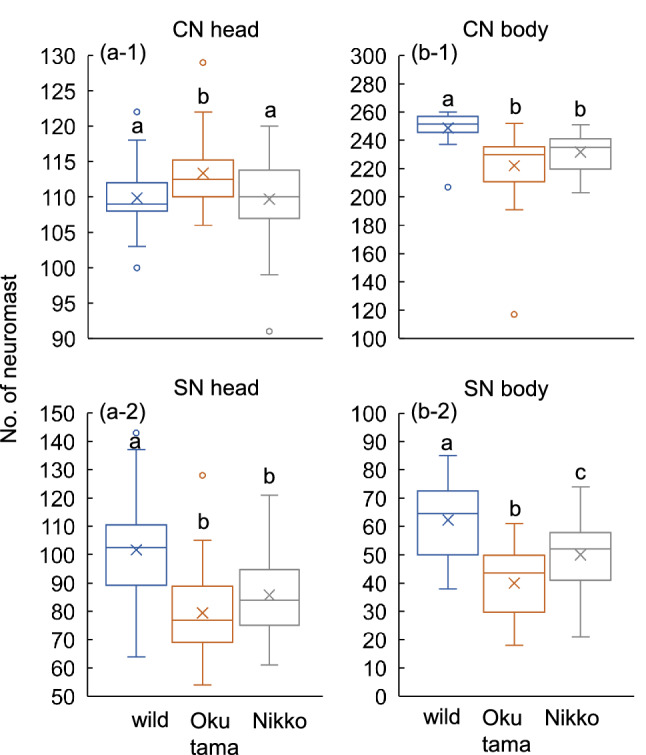
Comparisons of neuromast numbers (CNs and SNs) on the head and body of wild-caught *Oncorhynchus masou masou* and those reared in the Okutama and Nikko hatcheries. All fish originated from the Okutama fluvial population. Different letters (a, b, c) indicate significant differences detected by Scheffe’s test following univariate ANOVA (p < 0.05). Boxplots show 25, 50 and 75 percentiles, maximum and minimum values without considering outliers, and mean values (X).

We examined wild, F1 hatchery and captive-bred fishes from anadromous and fluvial populations (i.e., Shiribetsu and Okutama populations) of masu salmon for analysis of neuromast number of the lateral line system (Figs. 1, 2, 3 and 4, Supplementary Tables 1 and 2; see “Methods” for detail).

### Variations in neuromast numbers between populations

Neuromast numbers differed between the Shiribetsu and Okutama populations (MANOVA: df = 4, Wilk’s *λ* = 0.248, *F* = 41.80, p < 0.001) (Fig. 2). Univariate ANOVAs indicated that CNs being more numerous on the head in the Shiribetsu population (df = 1, *F* = 52.31, *p* < 0.001) (Fig. 2a-1) and SNs more numerous in the Okutama population (df = 1, *F* = 16.73, *p* < 0.001) (Fig. 2a-2). CNs and SNs were both more numerous on the body in the Shiribetsu population compared with the Okutama population (CN: df = 1, *F* = 30.20, *p* < 0.001; SN: df = 1, *F* = 54.52, *p* < 0.001).

### Shiribetsu population

Neuromast numbers differed among F1, F13 and wild fishes (MANOVA: df = 8, Wilk’s *λ* = 0.319, *F* = 16.16, *p* < 0.001) (Fig. 3). The number of CNs on the head of the F13 fish was lower than those on wild and F1 fishes (univariate ANOVA: df = 2, *F* = 23.56, *p* < 0.001) (Fig. 3a-1), whereas SN numbers on the head did not differ significantly among the three groups (univariate ANOVA: df = 2, *F* = 2.089, *p* = 0.130) (Fig. 3a-2). The number of neuromasts on the body also differed significantly among the wild, F1 and F13 fishes, although the patterns of differences among the three types differed slightly from those on the head (univariate ANOVA: CN: df = 2, *F* = 71.29, *p* < 0.001; SN: df = 2, *F* = 35.00, *p* < 0.001) (Fig. 3b-1 & -2). Overall, the total number of neuromasts (CN and SN) in F13 fish (490.9 ± 24.2) was ca. 11.7% lower than in wild-caught fish (555.9 ± 15.4) and ca. 9.9% lower than F1 fishes (545.0 ± 27.1).

### Okutama population

Neuromast numbers differed among Okutama hatchery, Nikko hatchery and wild fishes (MANOVA: df = 8, Wilk’s *λ* = 0.517, *F* = 9.973, *p* < 0.001) (Fig. 4). Although the number of CNs on the head of the Okutama hatchery individuals was greater than in the wild-caught and Nikko hatchery fishes (univariate ANOVA: df = 2, *F* = 5.135, *p* = 0.007) (Fig. 4a-1), the SN numbers on the head of the Okutama and Nikko hatchery fishes were both lower than on the wild fish (univariate ANOVA: df = 2, *F* = 16.45, *p* < 0.001) (Fig. 4a-2). CN numbers on the body of the Okutama and Nikko hatchery fishes (i.e., F 38 fishes) were lower than on the wild fish (univariate ANOVA: df = 2, *F* = 14.24, *p* < 0.001) (Fig. 4b-1). In addition to the significant lowness in the number of SNs on the body of the Okutama and Nikko hatchery fishes compared with that of wild-caught fish, a significant difference in the number existed between the Okutama and Nikko hatchery fishes (univariate ANOVA: df = 2, *F* = 22.50, *p* < 0.001) (Fig. 4b-2). Overall, the total number of neuromasts (CNs and SNs) in the Okutama (454.8 ± 42.5) and Nikko (477.1 ± 31.5) fishes were ca. 13.0% and 8.7% lower than in the wild-caught fish (522.5 ± 31.2), respectively. The total number of neuromasts were also differ between the Okutama (454.8 ± 42.5) and Nikko (477.1 ± 31.5) hatchery fishes.

## Discussion

This is the first study in which captive-bred fish have been shown as having undergone a reduction in neuromast numbers from that existing in the wild population, evidenced by a consideration of the unique neuromast numbers in each population of origin. In addition, F1 individuals whose parents had been obtained from natural environments had as similar numbers of neuromasts (CNs and SNs) as wild-caught fish. These results go a long way to confirming that the sensory organs of captive-bred fish had reduced in number through domestication.

Wild masu salmon were collected from two populations with differing life history strategies (i.e., anadromous and fluvial populations), and which were characterized by differing numbers of head and body neuromasts. Whether or not such neuromast conditions have adaptive significance is unclear, as the number of neuromasts of masu salmon may vary depending on the population. Brown et al. (2013)^14^ could not rule out the possibility that different population origins had influenced their finding of fewer neuromasts in captive-bred fish. However, the present results strongly suggest that domestication through captive breeding had resulted in a lowered number of neuromasts, regardless of population origin. Because the lateral line system is thought to influence many important behavioral features, for example, communication (including spawning), feeding and predator avoidance, rheotaxis and object entrainment, and schooling and swimming through sensing water movement^15^, a less than adequate number of neuromasts possibly decreases the survival potential and fitness of wild fish. However, because artificial ponds in hatcheries prevent intrusion of predators, and reared fish do not need to feed upon fast-moving prey, such fish can survive in captivity, even with a low number of neuromasts. Therefore, mean numbers of neuromasts may have gradually decreased through captive breeding. This study also found that the lowering of neuromast numbers differed between hatcheries (i.e., the Okutama and Nikko hatcheries), despite the origin of both captive-bred populations being the Okutama wild population. Because specific fish-rearing techniques adopted by hatchery staff may vary among hatcheries, different degrees of neuromast decrease between hatcheries may result. In addition, the absence of any apparent difference in number of neuromasts between wild and F1 fishes also suggests that a gradual decrease in neuromast numbers results from domestication (through prolonged captive breeding).

CN and SN numbers on the body both decreased in fishes from both populations following captive breeding. However, a decrease in neuromast numbers on the head was less obvious. In fact, SNs in the Shiribetsu population and CNs in the Okutama population did not decrease. Within the lateral line system as a whole, neuromasts on the head may be more important for detecting pellets thrown into the rearing ponds, as well as avoiding collision with the concrete wall of the raceway, than those on the body. Accordingly, neuromast numbers on the head may have been sustained, unlike neuromasts on the body, even following domestication, although these findings contradicted those of Brown et al.^14^, who reported lower SN numbers on the head of captive-bred steelheads, compared with wild individuals, and no significant difference in SN numbers on the body. To better understand the morphology of the lateral line system in captive-bred fishes, a broader study approach is needed, including case studies over a number of taxa and detail observations of neuromast conditions.

A decrease in sensory organs following captive breeding has also been reported in other taxa (e.g., the Lord Howe Island stick insect *Dryococelus australis*^6^). It is unsurprising that less sensitive (e.g., low numbers of sense organs) individuals can survive in rearing environments that lack predators and have abundant food supply. However, such individuals may show lower survival fitness than wild individuals in natural habitats. In fact, captive-bred masu salmon from both anadromous and fluvial wild populations were at one time released into the wild for commercial coastal fisheries and inland-water recreational fishing in Japan, but their survival rates tended to be low^16^. It has been well established that morphological and behavioral evolution occurs in captive-bred fishes (including salmonids) through domestication^17^, such changes being associated negatively with relative fitness^18^. The decrease in neuromast numbers demonstrated in this study may also be one of the causes of lowered survival of captive-bred fish returned to the natural environment^19,20^, and demonstrates the need for a broader understanding of factors necessary for successful stocking of captive-bred individuals in natural environments.

## Methods

### Materials

One hundred and ninety-eight young-of-the-year masu salmon were examined, having been obtained from the wild environment by electrofishing (Smith-Root Inc., Vancouver, WA, USA) with legal permissions from the Governors of Hokkaido and Tokyo, and from fish hatcheries in Japan. No wild fish was replenished to fish hatcheries for captive breeding. All specimens examined in the study were deposited in the National Museum of Nature and Science, Tsukuba, Japan.

#### Shiribetsu population

Wild fish (n = 30, 38.1–55.3 mm FL) were collected from three tributaries (Asase, Takinosawa and San’nosuke Rivers) of the Shiribetsu River system, Hokkaido, northern Japan in May–June 2018 (prior to the release of hatchery fish into the river system that year); F1 hatchery fish (see “Terminology” below; n = 30, 38.1 − 62.1 mm FL) from the Rankoshi fish hatchery (located at 42°46′41.7"N 140°26′11.6"E) of the Shiribetsu field station, Fisheries Resources Institute (formerly, Hokkaido National Fisheries Research Institute), Japan Fisheries Research and Education Agency (FRA) in May 2018; and captive-bred fish (n = 30, 43.7 − 71.8 mm FL) from the fish hatchery of the Nikko field station (located at 36°45′20.2"N 139°27′03.7"E), Fisheries Technology Institute (formerly, National Research Institute of Fisheries Science), FRA in May 2018. The captive-bred fish were approximately 13th generation descendants of the Shiribetsu River wild population, the first generation having been established in 1978/79, and subsequent generations every 3 years following. Captive-bred masu salmon have not been released for many years into the Shiribetsu River, so far as is known.

#### Okutama population

Wild fish (n = 30, 66.7–98.1 mm FL) were collected from the Kurasawa Valley, a tributary of the Nippara River (Okutama Area) of the Tama River system, Tokyo, Japan in November 2018; and captive-bred fish from the Irikawa fish hatchery of the Okutama Fish Farming Center, Tokyo Development Foundation for Agriculture, Forestry, and Fisheries (n = 30, 91.2–108.8 mm FL), via an fishery company in October 2018, and from the Nikko Field Station (n = 48; 42.4–139.2 mm FL) in May and December 2018. The captive-bred fish were mostly 38th generation descendants of the original upper Tama River system population, which had been maintained since 1944, with generation changes every 2 years. Part of the captive-bred population at the Okutama Fish Farming Center was transferred to the Nikko Field Station in 2010. Because no F1 hatchery masu salmon were present in the Tama River system, so far as is known, the neuromast numbers within the Okutama fluvial population were compared among the wild and captive-bred fish reared in the Okutama and Nikko hatcheries.

Wild fish from the Okutama fluvial population in the Kurasawa Valleywere collected above an unpassable (for salmonids) waterfall where captive-bred fish had not been released for many years.

### Neuromast counts

Canal (CN) and superficial (SN) neuromasts of each element of the lateral line system on both the head and body (see Fig. 1 and below) were counted on fish stained with DiAsp^21^ (see also Supplementary Fig. 1) and anesthetized (and euthanized) with ca. 0.1% solution of FA 100 (4-Allyl-2-methoxyphenol: eugenol).

### Terminology

The terminology of each element of the lateral line system of masu salmon follows Nakae & Hasegawa (2022^22^; see also Fig. 1). Neuromasts on the body comprised CNs in the trunk canal (TRC) and superficial neuromast groups of the predorsal (pd), trunk accessory (tra) and caudal fin (cf); neuromasts on the head comprised all other elements. The term “wild fish” in this study is applied to individuals born in the wild, but whose parental origins (i.e., wild or hatchery) were unclear. F1 hatchery fish indicates hatchery-reared individuals whose parents were individuals migrating from the sea to the Shiribetsu River for spawning, but whose origins (i.e., wild or hatchery) were unclear.

### Statistical analyses

Numbers of neuromasts (CNs and SNs) were recorded for the head, body of each fish. Multivariate Analysis of Variance (MANOVA) followed by univariate ANOVA (analysis of variance) which has number of CNs and SNs on head and body as dependent variables and source populations as independent variables were performed to compare the values for wild-caught fish between the Shiribetsu and Okutama populations.

Subsequently, MANOVAs followed by univariate ANOVA were performed to compare values (i.e., number of CNs and SNs on head and body) among wild fish, F1 and F13 hatchery fish from the Shiribetsu population (generation of fish as an independent variable), and wild fish, and Okutama and Nikko hatchery fish from the Okutama population (location of fish hatchery as an independent variable), respectively. For these analyses, univariate ANOVAs were followed by Scheffe’s test.

All numbers of neuromasts were log_10_-transformed for the statistical tests. The alpha level was set at 0.05. The SPSS version 24 (IBM Corp., Armonk, New York, USA) was used for performing the statistical tests.

### Ethical statement

The study protocol was approved by the animal ethics committee of National Museum of Nature and Science, and was conducted in strict adherence with guidelines for the care and use of research animals set out by the committee and in compliance with the ARRIVE guidelines v1.0^23^ (our experiments have done prior to ARRIVE guidelines v2.0^24^ published on 2020). Before the neuromasts count, the animals were euthanized with ca. 0.1% solution of FA 100.

## Supplementary Information

Supplementary Information.

## Data Availability

The raw data are available in supplementary tables.
