# Supplementary material for: Domestication of captive-bred masu salmon Oncorhynchus masou masou (Salmonidae) leads to a significant decrease in numbers of lateral line organs
Source: Sci Rep. 2022 Oct 6;12:16780. doi: 10.1038/s41598-022-21195-3 (PMC9537280; doi:10.1038/s41598-022-21195-3)
Supplement: Supplementary file 1 — Supplementary Information. [file 41598_2022_21195_MOESM1_ESM.pdf]

Domestication of captive-bred masu salmon *Oncorhynchus masou masou* (Salmonidae) leads to a significant decrease in numbers of lateral line organs

Masanori Nakae, Koh Hasegawa and Kouta Miyamoto

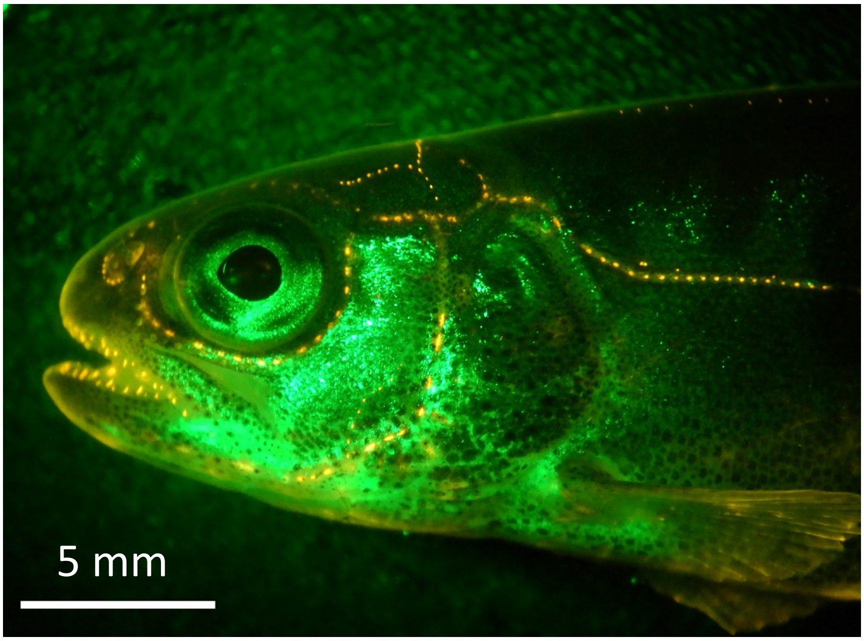

Supplementary Figure 1. Neuromasts of the lateral line system on the head in *Oncorhynchus masou masou*, stained by DiAsp.

Supplementary Table S1. Range and average (in parentheses) of neuromast numbers in each element of the lateral line system in three populations of anadromous *Oncorhynchus masou masou* . Neuromast numbers indicate those on both sides of the body. See Fig. 1 and accompanying legend for abbreviations.

Origin	n=	Size (mm in FL)	SOC	IOC	OTC	PRC	MDC	POC	STC	tTRC	TRC	ro	no	pio	po	ch	st	pd	tra	cf	Total CN	Total SN	Total
Wild	30	38.1–55.3	19–24	24–38	6–9	12–17	16–21	2–4	11–19	6–8	240–282	4–10	15–23	1–7	16–22	13–25	17–27	8–29	37–61	0–23	339–406	148–189	528–589
		(46.5)	(21.5)	(33.6)	(7.2)	(14.5)	(18.5)	(2.4)	(15.5)	(6.4)	(264.5)	(5.2)	(18.1)	(4.9)	(18.7)	(19.3)	(20.7)	(21.6)	(49.4)	(13.7)	(384.2)	(171.7)	(555.9)
Hatchery	30	38.1–62.1	17–24	29–37	5–8	13–17	15–21	2–4	10–20	6–8	220–278	4–7	16–23	3–7	14–25	13–26	14–27	13–29	28–56	0–29	326–403	129–198	455–595
		(51.4)	(21.6)	(33.1)	(6.8)	(14.3)	(18.4)	(2.4)	(15.8)	(6.4)	(261.5)	(4.6)	(19.7)	(5.0)	(18.7)	(19.3)	(20.9)	(22.3)	(43.9)	(10.2)	(380.4)	(164.6)	(545.0)
Captive-bred	30	43.7–71.8	22–24	31–35	7–8	14–15	14–20	2–3	14–17	6–8	234–250	7–7	17–20	5–8	18–23	21–23	11–27	17–29	29–46	18–18	311–366	112–179	434–537
		(62.0)	(21.4)	(32.2)	(6.5)	(14.2)	(15.6)	(2.0)	(13.5)	(6.3)	(232.7)	(4.6)	(17.2)	(4.5)	(18.1)	(17.5)	(22.8)	(18.7)	(31.8)	(11.3)	(344.4)	(146.5)	(490.9)

Supplementary Table S2. Range and average (in parentheses) of neuromast numbers in each element of the lateral line system in three populations of fluvial *Oncorhynchus masou masou* . Neuromast numbers indicate those on both sides of the body. See Fig. 1 and accompanying legend for abbreviations.

Origin	n=	Size (mm in FL)	SOC	IOC	OTC	PRC	MDC	POC	STC	tTRC	TRC	ro	no	pio	po	ch	st	pd	tra	cf	Total CN	Total SN	Total
Wild	30	66.7–98.1	17–25	27–37	4–8	12–16	14–20	2–2	10–17	6–6	207–260	0–11	16–24	2–7	13–33	6–40	6–40	6–32	23–47	1–23	317–382	113–222	438–580
		(84.4)	(19.7)	(31.5)	(6.4)	(13.8)	(16.7)	(2.0)	(13.7)	(6.0)	(248.7)	(6.8)	(20.2)	(4.3)	(23.4)	(23.7)	(23.3)	(18.4)	(34.7)	(9.1)	(358.5)	(163.9)	(522.5)
Captive-bred	30	91.2–108.8	16–26	27–38	4–8	13–16	14–19	1–4	10–16	5–9	117–252	1–13	10–22	0–6	5–26	13–30	6–42	2–28	1–37	0–34	227–375	81–189	318–546
Okutama		(99.8)	(20.5)	(32.8)	(6.3)	(14.1)	(16.8)	(2.0)	(13.8)	(6.9)	(222.0)	(5.6)	(16.1)	(3.3)	(14.3)	(21.9)	(18.3)	(14.5)	(15.2)	(10.3)	(335.3)	(119.5)	(454.8)
Captive-bred	48	42.4–139.2	15–23	29–38	4–7	11–15	11–20	2–3	8–18	4–9	203–251	2–14	11–32	0–9	10–28	10–32	12–39	1–24	15–39	0–31	303–366	83–177	386–531
Nikko		(63.6)	(19.3)	(33.3)	(6.0)	(13.9)	(15.3)	(2.0)	(13.6)	(6.4)	(231.7)	(4.5)	(15.8)	(4.4)	(18.0)	(19.4)	(23.7)	(13.7)	(26.5)	(9.9)	(341.4)	(135.8)	(477.1)

Supplementary Table S3. Body size (fork length) and neuromast numbers in each element of the lateral line system in *Oncorhynchus masou masou* examined in this study. Neuromast numbers indicate those on both sides of the body. Neuromasts of TRC, cf, pd and tra are categorized as those on the body. See Fig. 1 and accompanying legend for abbreviations.

ID	FL_mm	CN total	SN total	Grand Total	CN head	SN head	CN body	SN body	Head total	Body total	SOC	IOC	OTC	PRC	MDC	POC	STC	tTRC	TRC	ro	no	pio	ch	po	st	pd	tra	cf
Shiribetsu Wild 1	48.9	383	189	572	122	91	261	98	213	359	19	35	8	14	20	3	17	6	261	5	17	6	20	22	21	29	55	14
Shiribetsu Wild 2	46.2	379	167	546	122	83	257	84	205	341	23	33	8	16	17	2	17	6	257	5	15	4	18	21	20	26	47	11
Shiribetsu Wild 3	50.7	381	161	542	117	84	264	77	201	341	21	30	6	15	19	2	16	8	264	6	15	5	21	19	18	15	44	18
Shiribetsu Wild 4	45.9	403	179	582	125	85	278	94	210	372	21	38	7	15	19	2	17	6	278	6	16	4	17	20	22	22	53	19
Shiribetsu Wild 5	55.3	382	181	563	121	92	261	89	213	350	21	36	7	14	18	2	17	6	261	5	17	3	21	19	27	24	51	14
Shiribetsu Wild 6	44.6	387	174	561	116	94	271	80	210	351	21	34	8	14	18	2	13	6	271	4	17	5	22	22	24	21	48	11
Shiribetsu Wild 7	45.0	377	164	541	113	83	264	81	196	345	21	33	7	14	17	2	13	6	264	4	18	4	21	17	19	23	44	14
Shiribetsu Wild 8	46.6	385	185	570	122	91	263	94	213	357	22	34	8	14	18	2	18	6	263	5	22	6	19	18	21	21	54	19
Shiribetsu Wild 9	41.1	385	151	536	118	80	267	71	198	338	22	33	6	14	18	2	16	7	267	4	17	4	18	19	18	16	47	8
Shiribetsu Wild 10	38.1	372	160	532	116	81	256	79	197	335	23	32	6	14	18	2	15	6	256	4	20	5	16	19	17	20	45	14
Shiribetsu Wild 11	42.2	384	181	565	126	89	258	92	215	350	22	35	9	14	18	3	19	6	258	6	20	5	21	17	20	22	54	16
Shiribetsu Wild 12	49.6	393	176	569	118	96	275	80	214	355	21	34	8	14	21	2	12	6	275	5	20	4	23	19	25	17	45	18
Shiribetsu Wild 13	49.0	376	174	550	115	86	261	88	201	349	23	33	6	14	17	2	14	6	261	4	20	6	21	16	19	27	47	14
Shiribetsu Wild 14	46.8	391	168	559	127	83	264	85	210	349	19	35	9	17	19	3	17	8	264	4	19	6	19	17	18	21	42	22
Shiribetsu Wild 15	50.0	371	158	529	117	83	254	75	200	329	21	33	7	14	18	2	16	6	254	4	16	6	18	17	22	17	47	11
Shiribetsu Wild 16	48.5	378	189	567	117	94	261	95	211	356	22	32	6	14	18	2	16	7	261	6	18	6	22	22	20	25	50	20
Shiribetsu Wild 17	43.1	392	172	564	126	101	266	71	227	337	24	36	8	16	18	2	15	7	266	5	23	7	22	21	23	29	42	0
Shiribetsu Wild 18	43.8	382	181	563	120	83	262	98	203	360	22	31	8	14	20	3	16	6	262	4	20	6	16	18	19	28	55	15
Shiribetsu Wild 19	44.4	381	170	551	110	82	271	88	192	359	21	31	6	14	16	2	14	6	271	5	16	6	15	20	20	18	47	23
Shiribetsu Wild 20	45.9	390	168	558	121	86	269	82	207	351	22	34	6	16	18	2	17	6	269	4	18	5	19	19	21	20	47	15
Shiribetsu Wild 21	54.3	396	168	564	127	87	269	81	214	350	23	35	8	15	19	4	16	7	269	4	19	4	18	20	22	20	46	15
Shiribetsu Wild 22	51.6	386	159	545	123	90	263	69	213	332	22	33	6	16	18	4	18	6	263	6	18	5	20	17	24	16	45	8
Shiribetsu Wild 23	43.7	339	189	528	99	92	240	97	191	337	20	24	6	12	18	2	11	6	240	10	17	1	25	17	22	27	54	16
Shiribetsu Wild 24	46.3	390	171	561	124	89	266	82	213	348	21	35	8	16	18	3	16	7	266	7	19	4	19	19	21	22	51	9
Shiribetsu Wild 25	45.3	385	158	543	124	76	261	82	200	343	23	36	6	16	19	2	16	6	261	4	17	4	13	19	19	17	54	11
Shiribetsu Wild 26	44.5	401	169	570	120	80	281	89	200	370	20	36	8	14	18	3	15	6	281	6	16	5	17	16	20	24	57	8
Shiribetsu Wild 27	45.5	406	183	589	124	89	282	94	213	376	22	37	8	13	19	2	15	8	282	6	17	7	21	20	18	23	61	10
Shiribetsu Wild 28	52.4	391	177	568	124	84	267	93	208	360	21	34	8	15	21	4	14	7	267	6	19	4	18	16	21	22	55	16
Shiribetsu Wild 29	44.8	384	148	532	121	91	263	57	212	320	21	33	6	14	21	3	16	7	263	8	18	4	21	16	24	8	37	12
Shiribetsu Wild 30	39.7	375	182	557	116	84	259	98	200	357	20	34	9	14	18	2	13	6	259	5	18	7	19	18	17	29	59	10
Shiribetsu Hatchery 1	57	388	189	577	124	103	264	86	227	350	23	35	8	15	19	3	14	7	264	5	20	7	26	21	24	24	56	6
Shiribetsu Hatchery 2	57.8	403	192	595	125	102	278	90	227	368	24	32	6	14	21	2	20	6	278	6	18	4	24	25	25	27	53	10
Shiribetsu Hatchery 3	55.8	389	171	560	115	97	274	74	212	348	20	34	6	13	18	2	14	8	274	5	22	6	19	19	26	16	41	17
Shiribetsu Hatchery 4	53.9	386	173	559	118	86	268	87	204	355	20	34	6	14	19	2	16	7	268	4	22	4	22	16	18	20	55	12
Shiribetsu Hatchery 5	62.1	394	164	558	122	94	272	70	216	342	23	34	7	14	21	3	13	7	272	6	19	6	22	21	20	20	46	4
Shiribetsu Hatchery 6	55.6	384	171	555	121	91	263	80	212	343	20	35	8	15	19	2	16	6	263	4	18	3	24	15	27	23	41	16
Shiribetsu Hatchery 7	56.6	380	160	540	116	87	264	73	203	337	22	31	7	14	19	2	15	6	264	4	20	7	19	20	17	19	39	15
Shiribetsu Hatchery 8	51.6	394	169	563	120	91	274	78	211	352	20	36	7	16	18	3	14	6	274	6	22	3	19	19	22	27	41	10
Shiribetsu Hatchery 9	38.1	326	129	455	106	68	220	61	174	281	20	29	5	15	18	2	10	7	220	4	17	6	13	14	14	18	39	4
Shiribetsu Hatchery 10	51	390	148	538	128	86	262	62	214	324	22	34	7	14	20	4	20	7	262	4	17	4	17	21	23	17	45	0
Shiribetsu Hatchery 11	52.5	385	198	583	116	95	269	103	211	372	21	33	6	13	18	2	17	6	269	5	19	5	23	22	21	29	56	18
Shiribetsu Hatchery 12	54.4	387	152	539	113	80	274	72	193	346	21	31	7	14	19	2	13	6	274	4	21	6	17	15	17	22	45	5
Shiribetsu Hatchery 13	47.9	388	163	551	130	87	258	76	217	334	23	35	8	17	20	4	17	6	258	4	17	6	21	17	22	23	44	9
Shiribetsu Hatchery 14	51.6	376	158	534	118	86	258	72	204	330	21	35	6	14	16	3	17	6	258	4	22	4	18	18	20	25	42	5
Shiribetsu Hatchery 15	46.9	361	142	503	119	85	242	57	204	299	22	34	6	14	19	2	16	6	242	4	20	5	13	20	23	20	37	0
Shiribetsu Hatchery 16	48.8	369	160	529	117	84	252	76	201	328	22	32	8	14	16	3	16	6	252	4	23	4	17	16	20	27	44	5
Shiribetsu Hatchery 17	54	370	155	525	113	88	257	67	201	324	17	32	6	14	15	3	20	6	257	6	20	4	15	20	23	22	36	9
Shiribetsu Hatchery 18	52.6	378	148	526	116	95	262	53	211	315	21	32	7	14	17	4	15	6	262	5	23	5	19	20	23	13	28	12
Shiribetsu Hatchery 19	52.8	376	155	531	114	85	262	70	199	332	21	32	7	13	18	2	15	6	262	5	21	6	17	19	17	19	40	11
Shiribetsu Hatchery 20	44.8	375	160	535	124	93	251	67	217	318	24	32	7	15	19	2	19	6	251	5	21	4	24	17	22	20	40	7
Shiribetsu Hatchery 21	50.2	362	163	525	114	76	248	87	190	335	22	31	6	14	18	2	15	6	248	4	16	5	16	16	19	25	38	24
Shiribetsu Hatchery 22	50.3	378	151	529	123	87	255	64	210	319	23	35	8	15	20	2	14	6	255	4	19	6	23	17	18	25	39	0
Shiribetsu Hatchery 23	49.3	370	144	514	113	80	257	64	193	321	22	33	7	15	15	2	12	7	257	5	18	5	18	16	18	17	37	10
Shiribetsu Hatchery 24	49.2	383	179	562	119	93	264	86	212	350	23	31	8	14	18	2	16	7	264	4	20	5	19	19	26	24	46	16
Shiribetsu Hatchery 25	50.5	389	178	567	115	88	274	90	203	364	19	31	6	14	20	2	17	6	274	4	20	6	21	18	19	25	46	19
Shiribetsu Hatchery 26	50.2	384	163	547	119	81	265	82	200	347	21	33	6	14	18	3	17	7	265	4	21	5	16	17	18	26	49	7
Shiribetsu Hatchery 27	54.7	403	185	588	129	84	274	101	213	375	24	37	8	15	18	2	18	7	274	4	20	5	14	22	19	25	47	29
Shiribetsu Hatchery 28	46.8	388	165	553	123	91	265	74	214	339	24	34	6	14	20	2	16	7	265	4	17	6	23	19	22	20	41	13
Shiribetsu Hatchery 29	46.5	381	178	559	120	99	261	79	219	340	22	34	7	13	18	2	17	7	261	7	21	5	20	23	23	23	51	5
Shiribetsu Hatchery 30	48.2	375	175	550	117	83	258	92	200	350	22	31	8	14	18	2	16	6	258	4	18	4	19	18	20	28	55	9
Shiribetsu Captive-bred 1	64.9	344	143	487	110	79	234	64	189	298	22	31	7	14	14	2	14	6	234	7	17	5	21	18	11	17	29	18
Shiribetsu Captive-bred 2	57.4	358	179	537	113	96	245	83	209	328	24	33	6	14	15	2	13	6	245	4	19	5	21	21	26	21	46	16
Shiribetsu Captive-bred 3	68.3	345	149	494	109	81	236	68	190	304	22	32	5	14	14	2	14	6	236	4	18	4	14	16	25	17	36	15
Shiribetsu Captive-bred 4	62	366	168	534	116	84	250	84	200	334	21	35	6	14	17	2	15	6	250	4	16	4	14	19	27	29	43	12
Shiribetsu Captive-bred 5	63.1	326	133	459	107	89	219	44	196	263	21	33	6	14	13	2	12	6	219	5	19	5	15	19	26	17	16	11
Shiribetsu Captive-bred 6	62.5	346	155	501	111	94	235	61	205	296	21	32	7	14	15	2	14	6	235	6	19	6	15	23	25	17	31	13
Shiribetsu Captive-bred 7	55	358	163	521	115	91	243	72	206	315	23	34	6	15	13	3	15	6	243	5	17	5	19	22	23	19	41	12
Shiribetsu Captive-bred 8	57.9	350	154	504	115	86	235	68	201	303	21	32	6	14	17	2	17	6	235	5	16	5	17	21	22	26	25	17
Shiribetsu Captive-bred 9	53.3	311	134	445	110	83	201	51	193	252	22	31	7	14	14	2	14	6	201	3	15	5	14	22	24	17	27	7
Shiribetsu Captive-bred 10	56.4	320	143	463	106	78	214	65	184	279	22	30	6	14	14	2	12	6	214	5	16	5	15	18	19	15	37	13
Shiribetsu Captive-bred 11	62.6	335	154	489	108	88	227	66	196	293	22	31	6	15	15	2	11	6	227	5	19	4	19	21	20	20	36	10
Shiribetsu Captive-bred 12	61.7	348	154	502	109	93	239	61	202	300	20	31	8	15	15	2	12	6	239	4	19	4	20	20	26	22	30	9
Shiribetsu Captive-bred 13	63	352	140	492	116	82	236	58	198	294	21	35	6	14	17	2	14	7	236	4	17	6	15	16	24	18	32	8
Shiribetsu Captive-bred 14	71.8	351	159	510	112	84	239	75	196	314	19	31	6	15	18	2	14	7	239	5	16	3	19	21	20	22	41	12
Shiribetsu Captive-bred 15	52.3	337	129	466	112	80	225	49	192	274	20	32	6	15	15	2	16	6	225	5	16	5	14	17	23	16	23	10
Shiribetsu Captive-bred 16	56.9	356	141	497	114	85	242	56	199	298	22	32	6	15	16	2	15	6	242	3	18	5	19	16	24	16	23	17
Shiribetsu Captive-bred 17	63.8	341	141	482	110	92	231	49	202	280	21	31	6	13	19	2	10	8	231	5	19	3	23	17	25	14	24	11
Shiribetsu Captive-bred 18	57.5	351	150	501	117	88	234	62	205	296	21	32	8	15	20	2	13	6	234	5	18	6	20	15	24	18	31	13
Shiribetsu Captive-bred 19	62.5	320	153	473	108	83	212	70	191	282	20	31	5	14	15	2	14	7	212	5	14	4	20	16	24	20	39	11
Shiribetsu Captive-bred 20	67.1	345	143	488	114	92	231	51	206	282	22	33	6	14	15	2	16	6	231	5	18	4	23	17	25	14	31	6
Shiribetsu Captive-bred 21	71.4	362	149	511	113	80	249	69	193	318	22	33	7	15	15	2	13	6	249	4	13	5	17	19	22	23	32	14
Shiribetsu Captive-bred 22	70.5	359	150	509	111	88	248	62	199	310	20	31	7	14	17	2	14	6	248	4	19	4	20	19	22	18	29	15
Shiribetsu Captive-bred 23	70.2	359	143	502	111	77	248	66	188	314	21	35	6	14	15	2	12	6	248	5	16	1	14	20	21	17	34	15
Shiribetsu Captive-bred 24	43.7	322	112	434	111	64	211	48	175	259	22	33	8	14	14	2	12	6	211	4	14	3	15	7	21	11	32	5
Shiribetsu Captive-bred 25	67.4	350	141	491	115	83	235	58	198	293	22	34	8	14	16	2	13	6	235	4	19	6	15	16	23	27	27	4
Shiribetsu Captive-bred 26	62.6	342	143	485	109	81	233	62	190	295	22	30	6	14	17	2	11	7	233	4	17	4	18	12	26	17	36	9
Shiribetsu Captive-bred 27	70.3	356	147	503	118	89	238	58	207	296	22	34	7	14	18	2	13	8	238	6	16	3	23	18	23	22	25	11
Shiribetsu Captive-bred 28	51.1	316	127	443	110	81	206	46	191	252	23	32	6	14	13	2	14	6	206	4	16	5	13	19	24	16	27	3
Shiribetsu Captive-bred 29	68	346	154	500	107	90	239	64	197	303	20	29	8	14	16	2	12	6	239	4	20	8	14	18	26	18	36	10
Shiribetsu Captive-bred 30	64	360	145	505	115	80	245	65	195	310	21	33	6	14	17	2	15	7	245	4	20	3	20	19	14	17	36	12
Omutama Wild 1	87.5	374	139	513	115	90	259	49	205	308	21	32	7	14	18	2	15	6	259	6	22	6	22	25	9	24	23	2
Omutama Wild 2	74.7	351	149	500	108	103	243	46	211	289	19	31	7	14	18	2	11	6	243	9	23	4	20	23	24	14	25	7
Omutama Wild 3	91.9	354	160	514	106	100	248	60	206	308	18	27	7	14	17	2	15	6	248	6	17	4	27	24	22	22	31	7
Omutama Wild 4	84.0	353	160	513	108	89	245	71	197	316	19	31	6	14	17	2	13	6	245	4	16	4	17	20	28	22	30	19
Omutama Wild 5	92.2	371	184	555	114	111	257	73	225	330	19	33	7	14	18	2	15	6	257	10	23	5	31	28	14	16	43	14
Omutama Wild 6	69.7	317	121	438	110	83	207	38	193	245	18	31	7	14	16	2	16	6	207	4	18	5	21	19	16	9	25	4
Omutama Wild 7	98.1	358	222	580	104	137	254	85	241	339	18	30	6	14	15	2	13	6	254	8	23	5	40	29	32	22	43	20
Omutama Wild 8	89.4	355	158	513	106	89	249	69	195	318	21	31	5	13	16	2	12	6	249	5	19	6	27	26	6	18	44	7
Omutama Wild 9	95.9	363	203	566	116	127	247	76	243	323	21	35	6	14	18	2	14	6	247	8	21	6	33	24	35	24	35	17
Omutama Wild 10	89.3	368	171	539	109	97	259	74	206	333	21	29	6	14	18	2	13	6	259	4	21	6	21	25	20	23	44	7
Omutama Wild 11	70.1	366	135	501	113	79	253	56	192	309	22	30	6	14	16	2	17	6	253	6	20	5	11	19	18	24	23	9
Omutama Wild 12	85.9	361	184	545	109	116	252	68	225	320	21	29	6	14	18	2	13	6	252	8	23	5	24	28	28	25	33	10
Omutama Wild 13	96.1	363	172	535	112	119	251	53	231	304	19	33	8	14	16	2	14	6	251	10	22	5	29	23	30	17	28	8
Omutama Wild 14	86.4	361	207	568	112	143	249	64	255	313	20	34	6	13	17	2	14	6	249	8	24	6	35	30	40	24	35	5
Omutama Wild 15	89.7	318	173	491	109	105	209	68	214	277	23	30	7	13	16	2	12	6	209	11	19	6	26	28	15	27	35	6
Omutama Wild 16	78.3	359	133	492	100	80	259	53	180	312	17	30	6	14	15	2	10	6	259	7	17	4	8	18	26	6	31	16
Omutama Wild 17	75.5	362	144	506	103	96	259	48	199	307	19	32	6	12	14	2	12	6	259	6	24	2	19	25	20	15	32	1
Omutama Wild 18	90.4	367	198	565	118	121	249	77	239	326	25	35	6	13	16	2	15	6	249	8	21	3	34	33	22	21	41	15
Omutama Wild 19	72.4	367	113	480	108	64	259	49	172	308	20	34	7	13	16	2	10	6	259	4	16	2	6	24	12	8	30	11
Omutama Wild 20	87.0	382	175	557	122	109	260	66	231	326	20	37	6	14	20	2	17	6	260	9	21	6	26	28	19	22	36	8
Omutama Wild 21	78.3	351	149	500	109	102	242	47	211	289	19	31	8	14	15	2	14	6	242	8	21	3	27	20	23	14	31	2
Omutama Wild 22	74.4	345	172	517	108	107	237	65	215	302	19	31	6	14	16	2	14	6	237	10	18	4	27	19	29	16	39	10
Omutama Wild 23	88.0	360	166	526	107	104	253	62	211	315	19	33	4	14	17	2	12	6	253	5	22	3	23	19	32	18	32	12
Omutama Wild 24	85.6	353	155	508	111	89	242	66	200	308	17	32	7	14	17	2	16	6	242	6	19	2	29	18	15	19	42	5
Omutama Wild 25	80.4	365	134	499	110	70	255	64	180	319	19	29	6	16	17	2	15	6	255	0	18	3	15	13	21	14	47	3
Omutama Wild 26	93.7	363	181	544	106	107	257	74	213	331	19	31	6	14	16	2	12	6	257	10	20	2	22	24	29	21	45	8
Omutama Wild 27	83.0	359	142	501	109	95	250	47	204	297	20	31	7	14	15	2	14	6	250	3	20	6	23	18	25	9	35	3
Omutama Wild 28	66.7	353	149	502	108	100	245	49	208	294	20	28	7	14	17	2	14	6	245	5	19	7	24	18	27	12	25	12
Omutama Wild 29	89.6	374	181	555	115	106	259	75	221	334	20	33	6	14	19	2	15	6	259	4	19	2	26	25	30	32	41	2
Omutama Wild 30	86.7	363	188	551	110	112	253	76	222	329	19	32	7	13	18	2	13	6	253	11	20	2	19	28	32	15	38	23
Okutama Captive-bred 1	99.8	361	127	488	111	78	250	49	189	299	20	31	7	13	16	2	16	6	250	3	18	4	20	14	19	18	26	5
Okutama Captive-bred 2	95.6	333	95	428	120	65	213	30	185	243	25	32	6	14	19	2	15	7	213	7	10	4	22	5	17	20	8	2
Okutama Captive-bred 3	94.1	316	108	424	110	70	206	38	180	244	23	27	4	15	18	1	13	9	206	3	20	4	22	15	6	10	23	5
Okutama Captive-bred 4	98.8	342	115	457	116	69	226	46	185	272	24	31	5	14	16	2	15	9	226	6	14	6	15	12	16	20	11	15
Okutama Captive-bred 5	93.0	354	160	514	118	105	236	55	223	291	21	35	7	14	19	2	14	6	236	13	22	4	27	18	21	12	37	6
Okutama Captive-bred 6	98.2	334	100	434	114	69	220	31	183	251	24	30	7	13	17	2	14	7	220	4	13	4	19	12	17	16	7	8
Okutama Captive-bred 7	98.9	343	153	496	109	97	234	56	206	290	21	29	8	13	15	2	15	6	234	7	13	2	29	23	23	18	35	3
Okutama Captive-bred 8	97.7	375	135	510	129	89	246	46	218	292	26	38	6	15	18	2	16	8	246	2	13	4	25	17	28	14	9	23

Okutama Captive-bred 9	104.0	365	143	508	115	98	250	45	213	295	20	33	7	14	17	2	15	7	250	6	17	1	30	17	27	13	19	13
Okutama Captive-bred 10	103.2	310	123	433	115	71	195	52	186	247	24	33	6	14	17	1	13	7	195	5	15	6	19	8	18	15	20	17
Okutama Captive-bred 11	98.6	335	96	431	113	65	222	31	178	253	20	33	5	14	17	2	14	8	222	4	18	5	13	10	15	14	17	0
Okutama Captive-bred 12	103.9	336	105	441	110	79	226	26	189	252	19	35	6	14	15	1	13	7	226	1	17	5	18	15	23	7	8	11
Okutama Captive-bred 13	97.9	343	128	471	109	84	234	44	193	278	19	31	6	14	15	2	15	7	234	5	19	4	21	16	19	18	22	4
Okutama Captive-bred 14	99.4	325	99	424	114	81	211	18	195	229	21	32	7	14	18	2	12	8	211	6	15	4	20	12	24	13	3	2
Okutama Captive-bred 15	94.3	351	105	456	116	76	235	29	192	264	21	33	5	14	16	2	16	9	235	6	22	2	19	11	16	4	1	24
Okutama Captive-bred 16	100	341	122	463	106	67	235	55	173	290	17	32	6	14	14	2	15	6	235	3	14	4	20	12	14	28	10	17
Okutama Captive-bred 17	91.2	306	113	419	111	69	195	44	180	239	21	30	8	14	17	4	11	6	195	5	18	5	23	11	7	2	8	34
Okutama Captive-bred 18	103.8	303	135	438	112	92	191	43	204	234	18	32	8	14	17	2	15	6	191	10	12	3	27	18	22	17	26	0
Okutama Captive-bred 19	100.6	340	131	471	107	75	233	56	182	289	18	32	7	14	15	2	11	8	233	3	13	1	26	21	11	5	35	16
Okutama Captive-bred 20	104.8	227	91	318	110	69	117	22	179	139	19	36	7	15	16	2	10	5	117	4	18	2	22	10	13	7	11	4
Okutama Captive-bred 21	108.0	350	114	464	115	86	235	28	201	263	20	37	6	14	18	2	12	6	235	9	15	3	24	16	19	6	6	16
Okutama Captive-bred 22	103.6	327	133	460	114	89	213	44	203	257	21	34	7	14	15	2	14	7	213	5	15	2	25	17	25	12	28	4
Okutama Captive-bred 23	99.9	371	135	506	119	90	252	45	209	297	19	35	7	16	19	3	14	6	252	5	20	3	29	12	21	22	13	10
Okutama Captive-bred 24	95.4	311	81	392	112	54	199	27	166	226	19	35	5	16	16	3	12	6	199	2	16	0	20	8	8	21	2	4
Okutama Captive-bred 25	95.7	352	121	473	122	78	230	43	200	273	23	36	6	15	18	2	15	7	230	9	13	2	26	15	13	23	14	6
Okutama Captive-bred 26	105.6	345	101	446	108	67	237	34	175	271	16	32	7	13	18	2	12	8	237	5	13	3	15	14	17	18	7	9
Okutama Captive-bred 27	108.8	319	102	421	109	81	210	21	190	231	20	33	6	13	16	2	13	6	210	8	17	5	20	14	17	13	7	1
Okutama Captive-bred 28	106.4	346	121	467	111	69	235	52	180	287	19	31	6	14	18	2	15	6	235	4	15	4	16	14	16	20	18	14
Okutama Captive-bred 29	96.2	357	189	546	114	128	243	61	242	304	18	34	6	15	19	2	14	6	243	11	21	2	26	26	42	19	23	19
Okutama Captive-bred 30	97.9	341	104	445	111	74	230	30	185	260	19	33	5	14	16	2	14	8	230	6	18	2	18	16	14	11	1	18
Nikko Captive-bred 1	50	344	132	476	106	75	238	57	181	295	20	30	6	14	15	2	13	6	238	3	15	6	15	16	20	13	39	5
Nikko Captive-bred 2	53.6	350	138	488	113	84	237	54	197	291	19	35	6	14	17	2	14	6	237	4	18	4	18	20	20	11	29	14
Nikko Captive-bred 3	47.7	306	101	407	103	65	203	36	168	239	20	29	7	13	14	2	12	6	203	4	11	4	13	15	18	6	22	8
Nikko Captive-bred 4	59.6	336	143	479	111	89	225	54	200	279	20	36	4	13	16	2	14	6	225	4	13	6	20	21	25	16	38	0
Nikko Captive-bred 5	53	332	138	470	114	74	218	64	188	282	20	33	6	14	17	3	15	6	218	3	17	5	15	14	20	22	32	10
Nikko Captive-bred 6	48.3	324	131	455	108	63	216	68	171	284	22	30	6	14	14	2	12	8	216	5	12	4	14	13	15	21	39	8
Nikko Captive-bred 7	69	338	141	479	107	93	231	48	200	279	19	31	6	14	15	2	14	6	231	5	16	5	18	19	30	13	25	10
Nikko Captive-bred 8	42.4	337	137	474	103	73	234	64	176	298	21	30	5	15	13	2	11	6	234	4	13	3	17	16	20	14	32	18
Nikko Captive-bred 9	64.5	347	119	466	119	78	228	41	197	269	20	37	7	14	19	2	14	6	228	4	14	4	16	16	24	10	31	0
Nikko Captive-bred 10	47.8	336	134	470	118	95	218	39	213	257	21	35	6	15	20	2	13	6	218	5	19	8	22	20	21	14	15	10
Nikko Captive-bred 11	55.8	354	149	503	117	75	237	74	192	311	21	34	6	15	15	2	16	8	237	5	14	5	17	16	18	24	29	21
Nikko Captive-bred 12	60.1	320	129	449	116	81	204	48	197	252	21	34	6	15	17	2	15	6	204	2	12	9	19	16	23	17	25	6
Nikko Captive-bred 13	48.6	320	117	437	102	71	218	46	173	264	21	29	6	13	12	2	13	6	218	4	16	4	11	13	23	14	26	6
Nikko Captive-bred 14	50.9	343	110	453	105	67	238	43	172	281	19	31	5	13	14	2	13	8	238	4	14	3	13	16	17	13	24	6
Nikko Captive-bred 15	48.9	303	83	386	91	61	212	22	152	234	17	30	5	12	13	2	8	4	212	3	12	4	15	14	13	6	16	0
Nikko Captive-bred 16	111.2	353	116	469	112	82	241	34	194	275	20	37	6	14	16	2	13	4	241	4	15	4	18	17	24	10	20	4
Nikko Captive-bred 17	87.2	351	143	494	110	84	241	59	194	300	18	33	6	14	15	2	15	7	241	14	13	2	18	13	24	17	30	12
Nikko Captive-bred 18	108.3	353	144	497	117	95	236	49	212	285	20	36	6	14	15	2	17	7	236	4	18	7	19	19	28	11	32	6
Nikko Captive-bred 19	73.9	313	110	423	107	70	206	40	177	246	18	37	6	15	11	2	12	6	206	3	12	2	13	18	22	7	17	16
Nikko Captive-bred 20	85.5	353	172	525	115	104	238	68	219	306	21	35	7	14	16	2	12	8	238	4	15	4	28	24	29	18	30	20
Nikko Captive-bred 21	99.1	349	151	500	111	98	238	53	209	291	18	34	6	14	15	2	16	6	238	5	19	3	22	21	28	14	27	12
Nikko Captive-bred 22	99.0	348	152	500	107	83	241	69	190	310	18	34	4	14	17	2	12	6	241	3	17	0	20	17	26	14	39	16
Nikko Captive-bred 23	104.0	344	128	472	111	87	233	41	198	274	18	34	6	14	17	2	14	6	233	4	15	6	13	20	29	10	22	9
Nikko Captive-bred 24	98.0	338	150	488	111	99	227	51	210	278	18	34	6	15	16	2	11	9	227	4	14	5	27	19	30	13	34	4
Nikko Captive-bred 25	87.0	362	148	510	120	93	242	55	213	297	23	36	7	14	17	2	13	8	242	5	19	5	21	15	28	24	27	4
Nikko Captive-bred 26	106.2	345	161	506	103	113	242	48	216	290	16	32	6	14	15	2	14	4	242	8	20	5	28	20	32	13	24	11
Nikko Captive-bred 27	99.0	324	87	411	110	66	214	21	176	235	22	31	6	13	14	2	13	9	214	5	14	4	15	12	16	2	15	4
Nikko Captive-bred 28	86.2	343	142	485	108	92	235	50	200	285	18	30	6	15	14	2	18	5	235	5	17	4	22	17	27	10	28	12
Nikko Captive-bred 29	92.6	316	104	420	101	70	215	34	171	249	19	30	5	13	13	2	13	6	215	6	14	2	10	21	17	1	26	7
Nikko Captive-bred 30	97.2	361	132	493	116	80	245	52	196	297	21	35	6	15	18	2	12	7	245	5	12	6	22	21	14	17	28	7
Nikko Captive-bred 31	101.8	363	156	519	115	108	248	48	223	296	18	36	7	14	19	2	13	6	248	11	20	5	16	28	28	15	18	15
Nikko Captive-bred 32	89.0	351	166	517	106	109	245	57	215	302	17	33	6	14	14	2	14	6	245	4	18	4	30	25	28	16	23	18
Nikko Captive-bred 33	109.2	333	150	483	107	94	226	56	201	282	16	31	6	14	17	2	15	6	226	2	15	7	21	18	31	14	31	11
Nikko Captive-bred 34	73.4	322	130	452	108	78	214	52	186	266	17	32	6	14	16	3	12	8	214	5	15	3	15	21	19	17	20	15
Nikko Captive-bred 35	94.4	328	140	468	111	86	217	54	197	271	17	36	6	13	17	2	14	6	217	4	16	3	27	24	12	19	29	6
Nikko Captive-bred 36	103.6	355	112	467	112	77	243	35	189	278	22	35	6	14	12	2	14	7	243	4	21	5	15	10	22	12	19	4
Nikko Captive-bred 37	110.0	348	140	488	109	88	239	52	197	291	21	31	5	14	16	2	12	8	239	6	17	5	18	16	26	16	27	9
Nikko Captive-bred 38	104.4	361	131	492	112	72	249	59	184	308	20	34	6	14	16	2	14	6	249	5	15	3	16	17	16	13	30	16
Nikko Captive-bred 39	118.0	366	118	484	115	88	251	30	203	281	20	33	7	14	16	2	15	8	251	3	16	2	20	18	29	10	15	5
Nikko Captive-bred 40	103.7	339	124	463	104	84	235	40	188	275	21	32	5	11	14	2	13	6	235	3	16	7	18	17	23	12	21	7
Nikko Captive-bred 41	112.4	334	133	467	99	81	235	52	180	287	15	29	6	12	14	2	15	6	235	4	15	2	22	16	22	16	26	10
Nikko Captive-bred 42	113.6	354	151	505	109	92	245	59	201	304	20	34	6	14	14	2	12	7	245	3	13	3	24	20	29	23	25	11
Nikko Captive-bred 43	106.1	331	134	465	107	77	224	57	184	281	18	33	6	14	13	2	15	6	224	3	12	3	19	19	21	7	30	20
Nikko Captive-bred 44	109.0	361	169	530	120	108	241	61	228	302	23	36	7	14	16	2	15	7	241	4	19	5	28	21	31	9	21	31
Nikko Captive-bred 45	139.2	345	135	480	111	102	234	33	213	267	17	38	6	14	13	2	15	6	234	4	32	7	21	17	21	15	16	2
Nikko Captive-bred 46	126.0	354	177	531	107	121	247	56	228	303	17	35	6	13	14	2	14	6	247	4	16	3	32	27	39	10	33	13
Nikko Captive-bred 47	125.3	346	161	507	113	101	233	60	214	293	19	36	7	14	15	2	14	6	233	3	15	2	27	20	34	24	36	0
Nikko Captive-bred 48	85.5	351	148	499	108	90	243	58	198	301	17	34	6	14	16	2	13	6	243	4	18	7	23	13	25	15	29	14
